# Serum levels of sclerostin reflect altered bone microarchitecture in patients with hepatic cirrhosis

**DOI:** 10.1007/s00508-019-01595-8

**Published:** 2020-01-07

**Authors:** Robert Wakolbinger, Christian Muschitz, Jacqueline Wallwitz, Gerd Bodlaj, Xaver Feichtinger, Jakob E. Schanda, Heinrich Resch, Andreas Baierl, Peter Pietschmann

**Affiliations:** 1 Department of Physical and Rehabilitation Medicine, Danube Hospital – Social Medical Center East, Academic Teaching Hospital of the Medical University of Vienna, Langobardenstraße 122, 1220 Vienna, Austria; 2 Medical Department II — The VINFORCE Study Group, St. Vincent Hospital, Academic Teaching Hospital of the Medical University of Vienna, Stumpergasse 13, 1060 Vienna, Austria; 3 The Antibody Lab, Divischgasse 4, 1210 Vienna, Austria; 4 Division of Pharmacology, Department of Pharmacology, Physiology and Microbiology, Karl Landsteiner University of Health Sciences, Dr.-Karl-Dorrek-Straße 30, 3500 Krems, Austria; 5 AUVA Trauma Center Meidling, Kundratstraße 37, 1120 Vienna, Austria; 6 Karl Landsteiner Institute for Gastroenterology and Rheumatology, Stumpergasse 13, 1060 Vienna, Austria; 7 Department of Statistics and Operations Research, University of Vienna, Oskar-Morgenstern-Platz 1, 1090 Vienna, Austria; 8 Department of Pathophysiology and Allergy Research, Center for Pathophysiology, Infectiology and Immunology, Medical University of Vienna, Währinger Gürtel 18–20, 1090 Vienna, Austria

**Keywords:** Alcoholic liver cirrhosis, Trabecular number, Trabecular separation, Sandwich ELISA, Bone mineral density

## Abstract

**Background:**

Patients with hepatic cirrhosis are at increased risk of bone loss. Recent work on areal bone mineral density has reported contradictory findings. As the assessment of bone microarchitecture is complex, a search was made for correlations with new serum markers of bone turnover. Current data on serum sclerostin levels in patients with increased fracture risk are divergent and to date only one study has examined patients with hepatic cirrhosis. Therefore, the aim of this study was to evaluate serum sclerostin levels and to test for correlations with microarchitecture.

**Methods:**

This study was performed in 32 patients with recently diagnosed hepatic cirrhosis and 32 controls. The parameters of bone microarchitecture were assessed by high-resolution peripheral quantitative computed tomography. Sclerostin was detected via a new ELISA that detects the active receptor interaction site at loop 2 of the sclerostin core region.

**Results:**

Sclerostin levels were slightly, but not significantly lower in the patient group, compared to controls. In contrast, patients with alcoholic liver cirrhosis had significantly lower levels than the controls. A significant correlation with areal bone mineral density (BMD) and trabecular microarchitecture was observed in the patient group. However, there was hardly any correlation between sclerostin and bone microarchitecture in the controls.

**Conclusion:**

In hepatic cirrhosis, sclerostin is related to altered bone microarchitecture and lower areal BMD. In alcoholic liver disease, low sclerostin concentrations were seen.

## Introduction

In patients with hepatic cirrhosis areal bone mineral density (aBMD) is decreased especially at the lumbar spine [1, 2], whereas either decreased [3] or normal values [1, 2, 4] are observed at the hips. Due to technical limitations, aBMD alone can be insufficient to explain increased fracture risk [5] and bone microarchitecture can provide additional information. We recently investigated bone microarchitecture via high resolution peripheral quantitative computed tomography (HR-pQCT) [3]. As HR-pQCT is technically intensive, expensive and rarely available, serum bone turnover markers (BTMs) should be tested for predictive value. As standard serum markers of bone turnover do not reflect bone microarchitecture in hepatic cirrhosis [3], new markers need to be examined. Sclerostin, a glycoprotein secreted mainly by osteocytes, is a product of the *SOST* gene. It negatively regulates bone mass via binding to low-density lipoprotein receptor-related protein 5 and/or 6 (LRP5/6) and inhibits the Wnt/beta-catenin pathway, thereby decreasing bone formation [6]. To date, the only study investigating serum sclerostin in hepatic cirrhosis reported increased levels [7]. In patients at increased fracture risk, the results are divergent: Whereas some studies reported higher levels in postmenopausal osteoporotic women with fractures [8], diabetics [9] and alcoholics [10], others observed lower levels in geriatric patients with hip fractures [11]. These differences may result from methodological differences (fragments biasing measurements, different antibodies and therefore epitopes recognized, different assay constructions) [12].

A novel sclerostin ELISA was recently developed and launched (BI-20472, Biomedica, Vienna, Austria), which measures bioactive sclerostin by using a monoclonal antibody directed at the LRP5/6 binding region, thereby capturing all circulating sclerostin forms containing the free-receptor binding site. To ensure the reliability of this ELISA, it was validated in depth according to Food and Drug Administration quality standards. Therefore, the aforementioned limitations could be reduced, and the measured analyte specified.

This study shows validation data of the used ELISA and evaluated sclerostin in patients with hepatic cirrhosis, compared to matched healthy controls. The secondary objectives were first to test for correlations of sclerostin with trabecular and cortical bone microarchitecture and second, to examine relationships with serum markers of bone turnover.

## Methods

### Subjects

This study was conducted at the St. Vincent Hospital, an academic teaching hospital of the Medical University of Vienna. After approval by the St. Vincent Hospital ethics committee, patients with recently diagnosed hepatic cirrhosis were screened for eligibility. Written informed consent was obtained from all the patients and controls prior to any procedures. The definition of etiologies of hepatic cirrhosis, inclusion criteria and exclusion criteria have recently been reported [3]. The healthy controls (subject to the same exclusion criteria but no history or laboratory evidence of liver disease) were recruited from active and retired hospital staff.

### Serum bone turnover markers

The BTMs were obtained after overnight fasting between 8 and 10 a.m. at an ISO 9001 certified laboratory. Calcium, alkaline phosphatase, phosphorus, C‑terminal telopeptide of type I collagen (CTX), 25-OH vitamin D and intact parathyroid hormone (iPTH) were determined. Sclerostin serum concentration was determined via ELISA (BI-20472, Biomedica, Vienna, Austria) according to the manufacturer’s protocol. In contrast to conventional assays, this ELISA is designed to detect the active receptor interaction site at loop 2 of the sclerostin core region.

### Validation of sclerostin ELISA

Validation experiments of the sclerostin ELISA (BI-20472, Biomedica, Vienna, Austria) were performed according to FDA quality guidelines. Specificity was assessed with a commonly used procedure of signal competition with an at least 5‑fold surplus of liquid capture antibody and by epitope mapping of linear epitopes of the compiled antibodies with a peptide microarray (Pepperprint GmbH, Heidelberg, Germany). Additionally, the limit of detection (LOD), lower limit of quantification (LLOQ), intra-assay precision, sample parallelism and accuracy were assessed.

The specificity of the ELISA to the protein of interest is one of the most important characteristics. The monoclonal antibody used for capture is directed against the receptor interaction site and is 100% specific for sclerostin. The polyclonal, horseradish peroxidase-labelled detection antibody has several linear epitopes throughout the molecule determined by a custom-made microarray analysis. For the sandwich ELISA, the competition of endogenous (8 samples) and recombinant sclerostin showed a mean specificity of 100% (99–100%).

### Areal bone mineral density

Dual X‑ray absorptiometry (DXA) at the lumbar spine (L1–L4), non-dominant radius (except for previous fractures), total body and hip was assessed. Fractured vertebrae were excluded.

### Bone microarchitecture

The HR-pQCT (XtremeCT, SCANCO Medical, Brütisellen, Switzerland) measurements of the non-dominant (except for previous fracture) distal radius and distal tibia were performed while immobilized in a carbon-fiber cast. Cortical volumetric BMD, trabecular bone volume fraction (TbBV/TV), trabecular number (TbN), trabecular thickness (TbTh), trabecular separation (TbSp), cortical thickness (CtTh) and cortical porosity (CtPo) were measured [3].

### Statistics

Group differences were analyzed using two-sample t‑tests. Distributional assumptions were checked visually by quantile-quantile plots. Multiple linear regression models were estimated with sclerostin as an independent variable and measures of BTMs, liver-related biochemistry and disease severity as dependent variables in separate models. Models with either albumin or adjusted calcium as additional covariate were estimated to investigate whether these parameters explain variation in addition to sclerostin. Model fits were quantified by adjusted R^2^-values. All tests were two-sided and *p* values less than 0.05 were considered statistically significant. All statistical analyses were performed with the statistical software R, version 3.50 (R Development Core Team, Vienna, Austria).

### Hypothesis

The hypothesis was to test whether or not serum levels of sclerostin would be altered in patients with hepatic cirrhosis.

## Results

In this study 32 patients (including 12 women) and 32 matched healthy controls (including 12 women) were included. None of the subjects had received a specific treatment for osteoporosis or chronic liver disease. The patients and controls were of similar age (median 62 and 60 years, respectively), 16 patients had alcoholic liver disease (ALD), 8 viral hepatitis, 5 non-alcoholic fatty liver disease, 2 hemochromatosis and 1 autoimmune hepatitis. Age and body mass index did not differ between the patients and controls but alcohol intake was higher in the patients (4 units/day vs. 1 unit/day). None of the patients with ALD were abstinent.

Patients with ALD (63 years) and other etiologies (62 years) were of similar age. The percentages of males were similar for patients with ALD (69%) and controls (63%) but slightly lower for patients with other etiologies (56%). The Child-Pugh score was similar among the patients with ALD (5) and other etiologies (6).

### Sclerostin ELISA validation

The sensitivity of the assay was 1.9 pmol/l and the lower limit of quantification was 2.5 pmol/l (data not shown). The precision of the assay (coefficient of variation) varied from 1% to 7%. The dilution linearity (also called sample parallelism) should ascertain that the affinity of the antibodies to endogenous sclerostin is similar to the recombinant calibrator. The calculated recovery of 1 + 1 and 1 + 3 diluted samples was between 86% and 125% for serum, EDTA and citrate plasma and therefore within the standard of acceptance. To examine the accuracy, which describes the closeness of determined values to the true concentration of the analyte, recombinant sclerostin with known concentration was added to samples and the percentage of recovery was calculated. Recovery in the lower range was 76–111% and in the higher range was 82–95%.

### Serum sclerostin levels

The sclerostin levels of all patients were slightly but not significantly (*p* = 0.18) lower (108 pmol/l, range 91–153 pmol/l) than in the controls (133 pmol/l, 104–181 pmol/l). In ALD, the levels were significantly (*p* = 0.045) reduced to 96 pmol/l (66–154 pmol/l) compared to the controls, whereas patients with other etiologies (120 pmol/l, 107–153 pmol/l) did not significantly differ from either the controls (*p* = 0.94) or from patients with ALD (*p* = 0.091).

### Regression and multivariate analysis

In the patients, sclerostin correlated significantly with aBMD and microarchitecture. Furthermore, a positive correlation with the model of end stage liver disease (MELD) score was observed and a trend for alcohol intake (*p* = 0.078). The strongest relations were found for radial TbSp and TbN at both the radius and tibia (Tables 1 and 2).

**Table 1:** Linear regression analysis for sclerostin with serum markers of bone turnover, liver-related chemistry and disease severity

	Beta coefficient patients	Adjusted R^2^ patients	*p*-value patients	Adjusted R^2^ (incl. albumin) patients	*p*-value albumin patients	Adjusted R^2^ (incl. adjusted calcium) patients	*p*-value adjusted calcium patients	Beta coefficient controls	Adjusted R^2^ controls	*p*-value controls
Ionized calcium (mmol/l)	−158.68	−0.027	0.516	−0.078	0.815	−0.055	0.497	0.000	−0.040	0.706
Calcium (mmol/l)	−66.99	−0.006	0.376	0.391	**<0.001**	0.117	**0.030**	0.000	−0.033	0.865
Adjusted calcium (mmol/l)	22.46	−0.031	0.803	0.152	**0.011**	n.a.	n.a.	0.000	−0.034	0.882
Phosphorus (mmol/l)	58.33	0.002	0.313	−0.031	0.834	0.036	0.162	0.000	−0.032	0.743
Intact parathyroid hormone (pg/ml)	−0.45	0.022	0.202	0.073	0.115	−0.009	0.784	0.013	−0.037	0.867
25OH vitamin D (ng/ml)	−0.56	−0.014	0.453	0.055	0.084	−0.048	0.911	0.006	−0.034	0.885
Crosslaps (CTX) (ng/ml)	8.03	−0.034	0.850	0.094	**0.035**	−0.061	0.584	0.000	0.024	0.216
Alkaline phosphatase (U/l)	0.60	0.006	0.287	0.247	**0.003**	0.014	0.269	−0.099	0.051	0.120
Albumin (g/dl)	−0.13	0.017	0.225	n.a.	n.a.	0.191	**0.011**	−0.001	−0.027	0.643
Bilirubin (mg/dl)	−17.77	0.087	0.056	0.342	**0.001**	0.118	0.161	0.000	−0.033	0.800
Glutamate oxaloacetate transaminase (GOT, U/l)	17.41	−0.033	0.930	0.125	**0.017**	−0.068	0.921	0.021	−0.001	0.330
Glutamic pyruvic transaminase (GPT, U/l)	−0.03	−0.031	0.811	−0.066	0.865	−0.064	0.797	0.064	0.020	0.219
Gamma glutamyl transpeptidase (GGT, U/l)	−0.10	0.099	**0.045**	0.070	0.774	0.072	0.708	−0.172	0.048	0.123
Partial thromboplastin time (s)	−0.08	−0.014	0.450	0.094	**0.041**	−0.045	0.757	0.017	0.001	0.321
International normalized ratio	1.64	−0.009	0.397	0.228	**0.003**	0.040	0.123	−0.001	0.149	**0.020**
Creatinine (mg/dl)	35.89	−0.022	0.568	0.014	0.159	0.028	0.122	0.000	−0.033	0.865
Child-Pugh score	6.18	−0.001	0.331	0.667	**<0.001**	0.089	0.056	n.a.	n.a.	n.a.
Model of end stage liver disease (MELD) score	5.95	0.124	**0.027**	0.578	**<0.001**	0.278	**0.011**	n.a.	n.a.	n.a.

Beta reflects the regression coefficient and adjusted R^2^ the coefficient of determination (adjusted on the number of predictors)

In addition, albumin and adjusted calcium were included in covariate models. *P*‑values lower then 0.05 and thereby significant relationships are indicated as bold

*n.a.* not applicable

**Table 2:** Linear regression analysis for sclerostin with areal bone mineral density and trabecular and cortical microarchitecture

	Beta coefficient patients	Adjusted R^2^ patients	*p*-value patients	Adjusted R^2^ (incl. albumin) patients	*p*-value albumin patients	Adjusted R^2^ (incl. adjusted calcium) patients	*p*-value adjusted calcium patients	Beta coefficient controls	Adjusted R^2^ controls	*p*-value controls
Lumbar spine BMD (L1-4)	85.72	0.055	0.105	0.132	0.064	0.120	0.082	0.001	0.012	0.255
T‑score lumbar spine	8.90	0.038	0.145	0.156	**0.031**	0.086	0.119	0.008	0.044	0.138
Total hip BMD	107.52	0.120	**0.030**	0.136	0.222	0.163	0.120	0.000	−0.001	0.332
T‑score hip	13.42	0.093	**0.050**	0.130	0.142	0.128	0.148	0.003	−0.009	0.393
Total body BMD	112.70	0.103	**0.041**	0.264	**0.010**	0.140	0.141	0.001	0.151	**0.020**
Radius BMD	140.09	0.064	0.092	0.049	0.462	0.064	0.326	0.001	0.026	0.193
Radius cortical BMD (mg/cm^3^)	0.03	−0.032	0.827	−0.016	0.237	−0.060	0.670	0.155	−0.010	0.410
Radius trabecular bone volume fraction	562.07	0.146	**0.018**	0.328	**0.005**	0.190	0.117	0.000	0.119	**0.032**
Radius trabecular number (1/mm)	65.41	0.279	**0.001**	0.401	**0.012**	0.353	**0.044**	0.000	−0.031	0.764
Radius trabecular thickness (mm)	120.43	−0.033	0.890	0.005	0.155	−0.067	0.866	0.000	0.231	**0.004**
Radius trabecular separation (mm)	−116.84	0.268	**0.001**	0.373	**0.021**	0.363	**0.026**	0.000	−0.003	0.348
Radius cortical thickness (mm)	40.20	0.001	0.323	−0.016	0.482	−0.021	0.551	0.000	−0.028	0.668
Radius cortical porosity (%)	2.95	−0.018	0.513	−0.004	0.241	−0.053	0.946	−0.001	−0.031	0.738
Tibia cortical BMD (mg/cm^3^)	−0.03	−0.032	0.821	0.102	**0.027**	0.004	0.162	0.296	0.019	0.219
Tibia trabecular bone volume fraction	564.62	0.155	**0.015**	0.344	**0.004**	0.270	**0.023**	0.000	0.001	0.317
Tibia trabecular number (1/mm)	69.63	0.255	**0.002**	0.341	**0.034**	0.330	**0.046**	0.000	−0.034	0.959
Tibia trabecular thickness (mm)	694.05	−0.004	0.356	0.064	0.085	0.016	0.216	0.000	0.068	0.084
Tibia trabecular separation (mm)	−182.23	0.188	**0.008**	0.348	**0.007**	0.325	**0.013**	0.000	−0.031	0.756
Tibia cortical thickness (mm)	−8.17	−0.032	0.836	0.178	**0.006**	0.124	**0.018**	0.001	0.012	0.253
Tibia cortical porosity (%)	1.86	−0.003	0.350	−0.004	0.331	−0.037	0.916	−0.007	−0.018	0.487

*BMD* bone mineral density

**Fig. 1:**
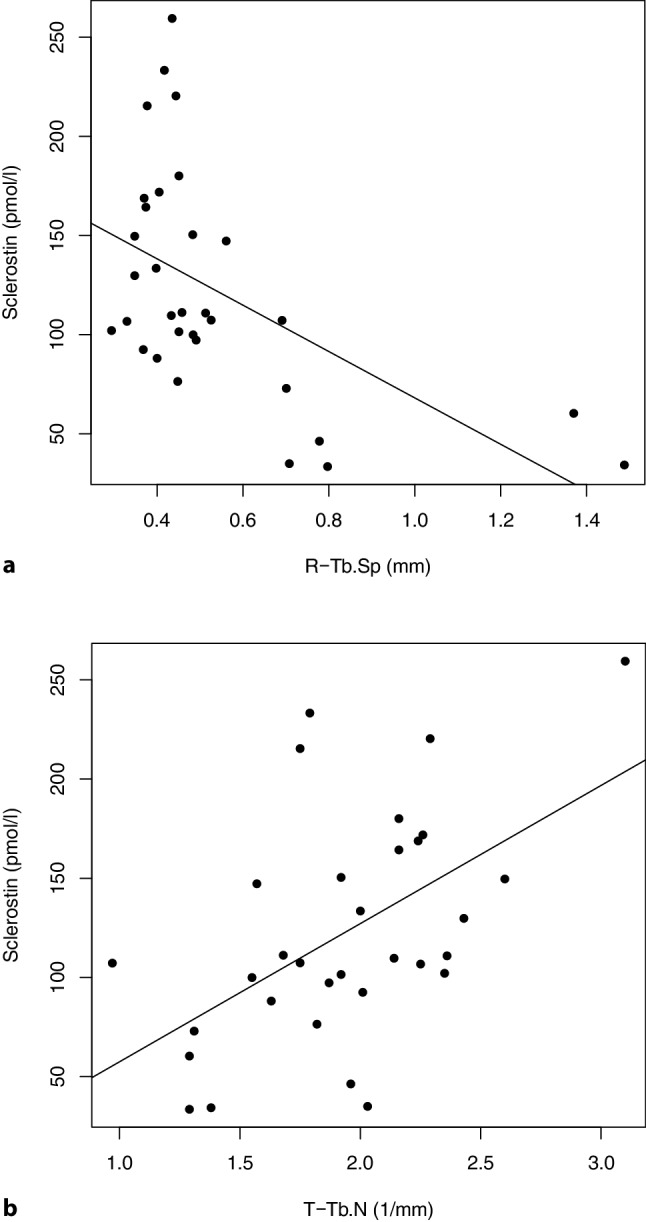
*Panel a*: the regression analysis demonstrates a significant (*p* = 0.001) inverse correlation between serum sclerostin and TbSp at the radius. *Panel b* reflects a significant (*p* = 0.002) direct correlation between serum sclerostin and TbN at the tibia

Fig. 1 demonstrates the relationship between sclerostin and parameters of trabecular microarchitecture.

Multivariate analysis demonstrated that the addition of albumin or adjusted calcium significantly enhanced the R^2^ value of sclerostin, especially for trabecular microarchitecture (Table 2). Albumin and adjusted calcium were not confounders and showed no correlation with microarchitecture without sclerostin (data not shown). Remarkably, in the controls, sclerostin showed hardly any correlation with bone microarchitecture, especially not at the weight-bearing tibia.

## Discussion

This study assessed the quality of sclerostin to explain bone microarchitecture in hepatic cirrhosis. We observed slightly lower sclerostin levels compared to controls. To date, a single previous study [7] has observed increased levels; however, neither BTMs, aBMD nor microarchitecture were assessed. Therefore, in our patients, aBMD could be lower due to advanced age. Moreover, we investigated Caucasians, whereas Rhee et al. [7] examined Koreans. Furthermore, most of their patients had viral hepatitis, whereas more than 50% of our patients had ALD. In a study on patients with primary biliary cholangitis (PBC), local expression of sclerostin in the bile ducts was reported, especially in early stages of the disease but declined in advanced disease stages [13]. Therefore, we assume that in our patients only a minor fraction of serum sclerostin is derived from hepatic production. Finally, within the characterization and validation of the ELISA used in our study we could show that it uniquely detects sclerostin molecules containing the LRP5/6 interaction site and therefore assumed to have bioactive function. As it is not completely understood which sclerostin molecules circulate (monomers vs. dimers [14], intact vs. fragments [15]), it is even more important to further characterize binding sites of utilized antibodies and therefore specify the detected analyte.

Within the technical validation of specificity, accuracy, intra-assay precision, limit of detection, lower limit of quantification and sample parallelism, we could show that the novel ELISA completely meets all quality standards comparable with other sclerostin assays on the market [16] but in contrast to those assays, the novel ELISA used in this study has clearly defined binding sites.

Sclerostin correlated with the MELD score, but neither the Child-Pugh nor the MELD scores improved the predictive value of sclerostin (data not shown). Albumin improved the R^2^ value of sclerostin for microarchitecture. These findings emphasize the presence of a liver-bone axis in terms of a strong relation between liver function and bone health. Especially albumin (synthetic function) together with sclerostin explained deranged bone structure in our patients. Similarly, in a large population-based outpatient study, lower albumin was independently associated with osteoporosis [17]. Rhee et al. [7] found higher sclerostin levels in advanced disease states and suggested lower hepatic clearance and altered concentrations of sex hormones to explain these findings. Similarly, patients with advanced ALD had higher sclerostin levels than in less pronounced states [10]. In contrast, those of our patients with ALD had significantly lower sclerostin levels, probably as alcohol promotes osteocyte apoptosis [18]. Moreover, maybe more sclerostin fragments are circulating and detected with their assay but not with our ELISA. Adjusted calcium improved R^2^ especially for trabecular microarchitecture, probably due to the correction for low albumin. Moreover, adjusted calcium was decreased in patients in whom we also observed deranged microarchitecture [3]; however, albumin and adjusted calcium did not correlate with sclerostin. Rhee et al. [7] did not test for relations with BTMs. Our patients’ sclerostin levels correlated with aBMD and microarchitecture. Similarly, in older men serum sclerostin [19] is related to microarchitecture and higher fracture risk. Lower serum sclerostin [11] and lower femoral sclerostin expression correlated with impaired trabecular microarchitecture in patients with hip fractures [20].

Sclerostin is produced predominantly by osteocytes. As low sclerostin expression is related to low bone volume and number of osteocytes per volume [21], lower trabecular bone volume and/or increased osteocyte apoptosis could explain the lower sclerostin levels in our patients. Alcoholism promotes osteocyte apoptosis, as demonstrated in a rat model [22]. In addition, in bone samples of patients with hepatic cirrhosis of various etiologies, a decreased number of osteocytes was reported [23]; however, bone histomorphometry was not available in our patients and therefore, further studies are required to confirm our assumption. In contrast, sclerostin levels could be decreased due to a rescue mechanism. A study on male idiopathic osteoporosis reported that lower sclerostin expression could reflect an autoregulatory promotion of bone formation [24]. As osteoblast activity is reduced in hepatic cirrhosis [25], this mechanism could act as an attempt to preserve osteogenesis. The relatively small sample size is a limitation of our study. The possibility exists that in a higher number of subjects some differences in serum sclerostin concentrations could become significant. In conclusion, serum sclerostin levels reflect deterioration of bone microarchitecture and osteocyte function in patients with hepatic cirrhosis. Patients with ALD had significantly lower sclerostin levels compared to other etiologies.

## Abbreviations

aBMD: Areal bone mineral density
ALD: Alcoholic liver disease
BTM: Bone turnover markers
CTX: C‑terminal telopeptide of type I collagen
CtPo: Cortical porosity
CtTh: Cortical thickness
DXA: Dual X‑ray absorptiometry
ELISA: Enzyme-linked immunosorbent assay
HR-pQCT: High resolution peripheral quantitative computed tomography
iPTH: Intact parathyroid hormone
MELD: Model of end stage liver disease
TbN: Trabecular number
TbSp: Trabecular separation
TbTh: trabecular thickness
TbBV/TV: Trabecular bone volume fraction
